# Comparison of iron isomaltoside ferumoxytol with iron sucrose for iron deficiency anemia: a meta-analysis of randomized controlled trials

**DOI:** 10.4314/ahs.v23i3.25

**Published:** 2023-09

**Authors:** Lunbo Shi, Yan Zhao, Aihua Rao

**Affiliations:** Department of hematological oncology chemotherapy, Fenghua District People's Hospital of Ningbo, Zhejiang, China

**Keywords:** Iron isomaltoside ferumoxytol, iron sucrose, iron deficiency anemia, randomized controlled trials

## Abstract

**Introduction:**

The efficacy of iron isomaltoside ferumoxytol versus iron sucrose to treat iron deficiency anemia remains controversial. We conduct this meta-analysis to explore the influence of iron isomaltoside ferumoxytol versus iron sucrose on iron deficiency anemia.

**Methods:**

We have searched PubMed, EMbase, Web of science, EBSCO, and Cochrane library databases through March 2021 for randomized controlled trials (RCTs) assessing the effect of iron isomaltoside ferumoxytol versus iron sucrose on iron deficiency anemia. Meta-analysis was performed using the random-effect model.

**Results:**

Four RCTs involving 3892 patients were included in the meta-analysis. Overall, compared with iron sucrose for iron deficiency anemia, iron isomaltoside showed similar change of Hb (SMD=0.14; 95% CI=-0.07 to 0.35; P=0.18), Hb responder (SMD=1.41; 95% CI=0.71 to 2.81; P=0.33), serum ferritin (SMD=15.13; 95% CI=-23.45 to 53.71; P=0.44), and transferrin saturation (SMD=1.20; 95% CI=-1.08 to 3.47; P=0.30). However, iron isomaltoside further improved serum-ferritin at week 2 than iron sucrose (SMD=204.79; 95% CI=38.23 to 371.35; P=0.02).

**Conclusions:**

Iron isomaltoside ferumoxytol showed comparable efficacy to iron sucrose for the treatment of iron deficiency anemia.

## Introduction

Iron deficiency anemia has become widespread prevalence in clinical work, and is mainly caused by gastrointestinal diseases, chronic kidney diseases, cancers, chronic heart failure, inflammatory bowel disease and bariatric procedures etc 1-5. The pathophysiological processes of anemia include blood loss, malnutrition, malabsorption of iron and impaired utilization of endogenous iron 6-9. Iron deficiency anemia may seriously impair patients' health-related quality of life, as evidenced by the reduced ability to work, fatigue and impaired physical and/or cognitive functioning 10-12.

Oral iron supplementation is widely accepted as the first-line treatment for iron deficiency anemia, but some patients need the intravenous iron as the preferred option when oral iron is ineffective or not tolerated, limited absorption, lack of adherence, intolerance or insufficient 13, 14. Intravenous iron may help improve iron correction with better adherence, fewer visit to the medical practitioner and greater convenience. Iron isomaltoside, also called ferric derisomaltose, is one novel intravenous iron formulation consisting of iron and a carbohydrate moiety where the iron is tightly bound in a matrix structure. Its matrix structure affords a controlled and slow release of iron to iron-binding proteins, avoiding potential toxicity from release of labile iron 15. Previous studies demonstrated good safety and efficacy of iron isomaltoside in different populations 16-18.

However, the efficacy and safety of iron isomaltoside ferumoxytol versus iron sucrose on iron deficiency anemia has not been well established. Recently, several studies on the topic have been published, and the results were conflicting 19-21. This meta-analysis of RCTs aimed to compare the efficacy and safety of iron isomaltoside ferumoxytol versus iron sucrose on iron deficiency anemia.

## Materials and methods

Ethical approval and patient consent were not required because this was a systematic review and meta-analysis of previously published studies. This meta-analysis was conducted and reported in adherence to PRISMA (Preferred Reporting Items for Systematic Reviews and Meta-Analyses) 22. The study protocol has not been published before.

### Search strategy and study selection

Two investigators have independently searched the following databases (inception to March 2021): PubMed, EMbase, Web of science, EBSCO, and Cochrane library databases. The electronic search strategy was conducted using the following keywords: “iron isomaltoside ferumoxytol” AND “iron sucrose” AND “anemia”. Two independent reviewers selected the eligible studies and checked the reference lists of the screened full-text studies to identify other potentially eligible trials.

The inclusive selection criteria were as follows: (i) patients are diagnosed with iron deficiency anemia; (ii) intervention treatment is iron isomaltoside ferumoxytol versus iron sucrose; (iii) study design is RCT.

### Data extraction and outcome measures

We extracted the following information: author, number of patients, age, female, hemoglobin (Hgb) level, transferrin saturation and detail methods in each group etc. Data were extracted independently by two investigators, and discrepancies were resolved by consensus. The primary outcome was Hb change. Secondary outcomes included Hb responder, serum ferritin, transferrin saturation, serum-ferritin at week 2, and adverse events.

### Quality assessment in individual studies

Methodological quality of the included studies was independently evaluated using the modified Jadad scale 23. There were three items for Jadad scale: randomization (0-2 points), blinding (0-2 points), dropouts and withdrawals (0-1 points). The score of Jadad Scale varied from 0 to 5 points. An article with Jadad score≤2 was considered to be of low quality. If the Jadad score≥3, the study was thought to be of high quality 24.

### Statistical analysis

We estimated the standard mean difference (SMD) with 95% confidence interval (CI) for continuous outcomes (Hb change, Hb responder, serum ferritin, transferrin saturation, and serum-ferritin at week 2) and odd ratio (OR) with 95% CIs for dichotomous outcomes (adverse events). The random-effects model was used regardless of heterogeneity. Heterogeneity was reported using the I2 statistic, and I2 > 50% indicates significant heterogeneity 25. Whenever significant heterogeneity was present, we searched for potential sources of heterogeneity via omitting one study in turn for the meta-analysis or performing subgroup analysis. Publication bias was not evaluated because of the limited number (<10) of included studies. All statistical analyses were performed using Review Manager Version 5.3 (The Cochrane Collaboration, Software Update, Oxford, UK).

## Results

### Literature search, study characteristics and quality assessment

**Figure 1:**
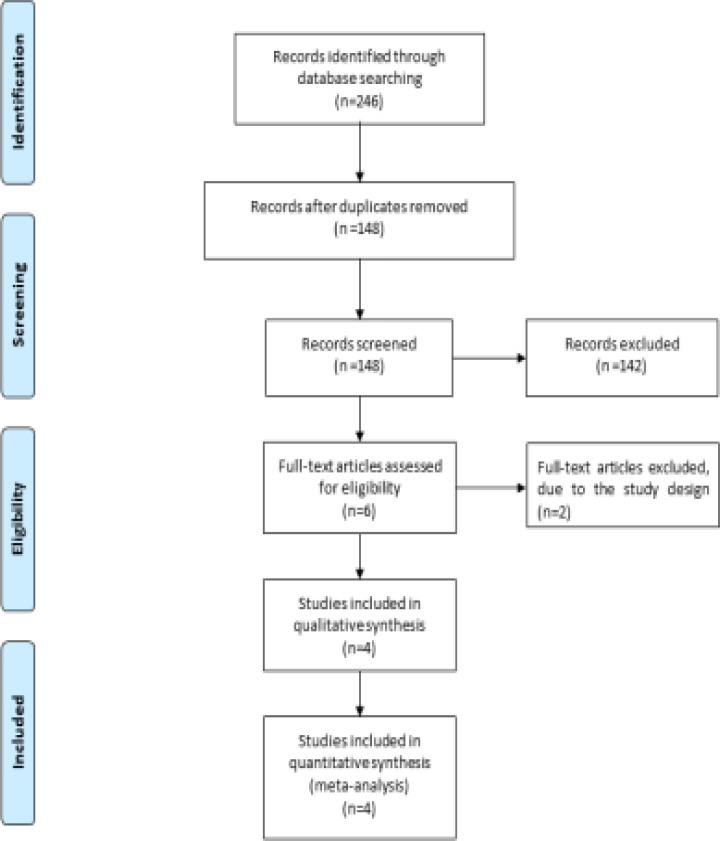
Flow diagram of study searching and selection process

A detailed flowchart of the search and selection results was shown in Figure 1. 249 potentially relevant articles were initially identified. 98 duplicates and 142 papers after checking the titles/abstracts were excluded. Two studies were removed because of the study design 16, 18, and four RCTs were ultimately included in the meta-analysis 19-21, 26.

The baseline characteristics of four eligible RCTs in the meta-analysis were summarized in Table 1. The four studies were published between 2015 and 2021, and sample sizes ranged from 351 to 1538 with a total of 3892. Iron isomaltoside was administered at a single dose of 1000 mg, while iron sucrose was administered at the dose of 100 or 200 mg several times. The causes of iron deficiency anemia included cancer, gastrointestinal disorders and chronic kidney disease etc.

**Table 1:** Characteristics of included studies

NO.	Author	Iron isomaltoside group						Iron sucrose group						Cause	Follow-up time	Jada scores
		Number	Age (years)	Female (n)	Hgb level (g/dL)	Transferrin saturation (%)	Methods	Number	Age (years)	Female (n)	Hgb level (g/dL)	Transferrin saturation (%)	Methods			
1	Bhandari 2021	1027	68.3 (12.3)	633	9.66 (1.14)	18.51 (29.23)	a single dose of 1000 mg iron isomaltoside 1000/ferric derisomaltose	511	69.3 (12.3)	329	9.71 (1.12)	17.44 (11.78)	iron sucrose administered as 200 mg IV injections up to five times within a 2-week period.	non-dialysis-dependent chronic kidney disease	8 weeks	5
2	Auerbach 2019	1009	44.1 (14.8)	892	9.25 (1.28)	7.43 (10.93)	a single dose of 1000 mg iron isomaltoside 1000/ferric derisomaltose	503	43.8 (14.4)	456	9.17 (1.27)	6.69 (7.44)	iron sucrose administered as 200 mg intravenous injections, up to five times	mixed etiologies	8 weeks	4
3	Derman 2017	330	49 (16)	297	-	-	a single dose of 1000 mg iron isomaltoside	161	47 (15)	146	-	-	an infusion of 200 mg over approximately 30 minutes up to twice weekly according to Ganzoni formula	mixed etiologies	5 weeks	4
4	Bhandari 2015	234	233 (60.13)	76	11.2 (0.66)	21.6 (5.95)	iron isomaltoside 1000	117	117 (59.5)	43	11.0 (0.76)	22.6 (6.76)	iron sucrose in split doses of 100 mg at baseline and 200 mg each at weeks 2 and 4	hemodialysis-chronic kidney disease	6 weeks	4

Among the four studies included here, three studies reported Hb change 19-21, 26, two studies reported Hb responder 19, 21, three studies reported serum ferritin, transferrin saturation and serum-ferritin at week 2 19, 21, 26, and three studies reported adverse events 20, 21, 26. Jadad scores of the four included studies varied from 4 to 5, and all four studies were considered to be high-quality ones according to quality assessment.

### Primary outcomes: Hb change

**Figure 2:**
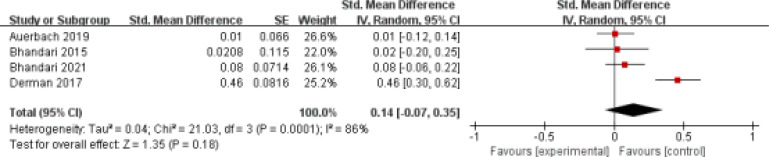
Forest plot for the meta-analysis of Hb change

This outcome data was analyzed with the random-effects model, and the pooled estimate of the four included RCTs suggested that compared to iron sucrose for anemia, iron isomaltoside showed similar change of Hb (SMD=0.14; 95% CI=-0.07 to 0.35; P=0.18), with significant heterogeneity among the studies (I^2^=86%, heterogeneity P=0.0001, Figure 2).

### Sensitivity analysis

Signiant heterogeneity was observed among the included studies for Hb change. As shown in Figure 2, the study conducted by Derman showed results that were almost out of range of the others and probably contributed to the heterogeneity 21. After excluding this study, the results suggested that iron sucrose and iron isomaltoside still resulted in comparable improvement in Hb for anemia (SMD=0; 95% CI=-0.16 to 0.16; P=0.44), and no heterogeneity remained (I^2^=0, P=0.87).

### Secondary outcomes

**Figure 3:**
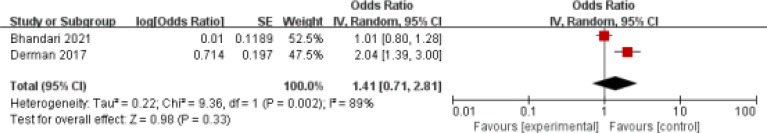
Forest plot for the meta-analysis of Hb responder

**Figure 4:**
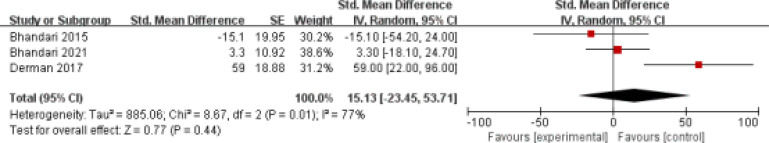
Forest plot for the meta-analysis of serum ferritin

**Figure 5:**
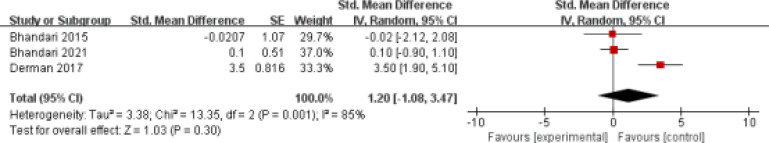
Forest plot for the meta-analysis of transferrin saturation

**Figure 6:**
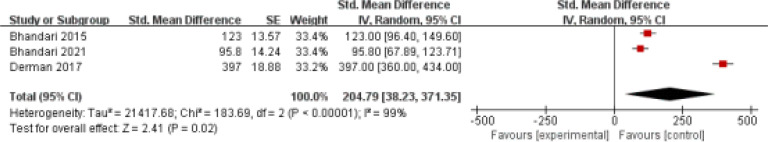
Forest plot for the meta-analysis of serum-ferritin at week 2

**Figure 7:**
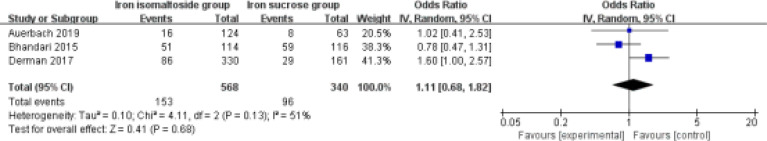
Forest plot for the meta-analysis of adverse events

Compared to iron sucrose for anemia, iron isomaltoside demonstrated comparable Hb responder (SMD=1.41; 95% CI=0.71 to 2.81; P=0.33; Figure 3), serum ferritin (SMD=15.13; 95% CI=-23.45 to 53.71; P=0.44; Figure 4), transferrin saturation (SMD=1.20; 95% CI=-1.08 to 3.47; P=0.30; Figure 5). However, iron isomaltoside was associated with significantly improved serum-ferritin at week 2 than iron sucrose (SMD=204.79; 95% CI=38.23 to 371.35; P=0.02; Figure 6). The incidence of adverse events was similar between two groups (OR=1.11; 95% CI=0.68 to 1.82; P=0.68; Figure 7).

## Discussion

Oral iron supplementation requires at least 2–3 weeks to increase the Hgb concentrations, up to 2 months to achieve normal values, and at least 6 months to replenish iron stores completely 27, 28. Intravenous iron is recommended to be more effective and better tolerated than oral iron, and is regarded as the preferred selection in many patients with iron deficiency anemia 29-31. The potential ability of intravenous iron to treat iron deficiency anemia has been demonstrated in patients with chronic kidney disease, abnormal uterine bleeding, pregnancy, postpartum anemia, cancer and gastrointestinal disorders 32-35.

Our meta-analysis confirmed that single dose of intravenous iron isomaltoside ferumoxytol and repeated doses of oral iron sucrose resulted in comparable Hb change, serum ferritin and transferrin saturation for the treatment of iron deficiency anemia. Due to the matrix structure of iron isomaltoside ferumoxytol, it can load high dose of iron and support a controlled and slow release of iron to iron-binding proteins 15, 36. The efficacy and safety of iron isomaltoside ferumoxytol is confirmed in this meta-analysis, as evidenced by no increase in adverse events compared to oral iron sucrose.

The ideal intravenous iron product is expected to allow iron correction and improvement in Hb in a single visit with a short infusion time and minimal side effects. Our meta-analysis suggests that a signal dose of iron isomaltoside ferumoxytol can promote a rapid improvement in serum-ferritin at week 2 than iron sucrose (SMD=204.79; 95% CI=38.23 to 371.35; P=0.02), which is very crucial for the patients requiring fast and large dose of iron supplementation. In addition, one RCT with 8 weeks of follow-up time, the incidence of cardiovascular adverse events was significantly lower in the iron isomaltoside ferumoxytol group compared with the iron sucrose group (4.1% versus 6.9%; P = 0.025), indicating the potential benefit of iron isomaltoside ferumoxytol to protect cardiovascular function 19.

Regarding the sensitivity analysis, there is significant heterogeneity for Hb change. After excluding the conducted by Derman with just five weeks of follow-up 21, no heterogeneity remained and iron sucrose and iron isomaltoside still have comparable improvement in Hb (P=0.44). These suggest iron isomaltoside may produce better efficacy in short time than that in relatively longer time, which is consistent with rapid improvement of serum-ferritin at week 2 than iron sucrose in this meta-analysis.

This meta-analysis has several potential limitations. Firstly, our analysis is based on only four RCTs and more RCTs with large samples should be conducted to confirm this issue. Secondly, significant heterogeneity is observed for the aalysis of Hb, which may be caused by the different follow-up time and patient population. Thirdly, iron deficiency anemia is caused by different diseases, which may affect the pooling results.

## Conclusions

Iron isomaltoside is effective and safe to treat iron deficiency anemia, and may benefit to reduce the risk of cardiovascular events.
